# POTENTIAL BENEFITS OF GROUP EXERCISE ON CANCER-RELATED LONELINESS AMONG MEN ENROLLED IN THE GET FIT PROSTATE TRIAL

**DOI:** 10.1093/geroni/igae098.2315

**Published:** 2024-12-31

**Authors:** Kerri Winters-Stone, Sydnee Stoyles, Nathan Dieckmann, Karen Lyons, Mary Crisafio, Arthur Hung, Julie Graff, Alexandra Sokolova

**Affiliations:** Oregon Health & Science University, Portland, Oregon, United States; Oregon Health & Science University, Portland, Oregon, United States; Oregon Health & Science University, Portland, Oregon, United States; Boston College, Chestnut Hill, Massachusetts, United States; Oregon Health & Science University, Portland, Oregon, United States; Oregon Health & Science University, Portland, Oregon, United States; Oregon Health & Science University, Portland, Oregon, United States; Oregon Health & Science University, Portland, Oregon, United States

## Abstract

Men with prostate cancer may experience cancer-related loneliness (CRL) that impacts their mental and physical health, yet research in this area remains sparse. We explored the relationship between cancer-related loneliness, anxiety, depressive symptoms, social and physical functioning and the potential benefits of group exercise in a subset of men with prostate cancer participating in a clinical exercise trial. We added the CRL questionnaire to measures of anxiety (PROMIS), depressive symptoms (CES-D) and social and physical function (QLQC30 subscales) mid-way through the GET FIT Prostate trial (NCT03741335). 145 men (mean age: 71.5 +/- 7.3 yrs) completed measures prior to participating in one of three online supervised group exercise programs for 6 months and another 68 also completed measures post-intervention. While exercise modalities differed among the three groups, all men participated in 15 minutes of semi-structured social time immediately prior to the start of their one-hour exercise class. 70% of men reported some level of CRL at baseline. CRL was significantly (p< 0.01) and positively associated with higher anxiety (r=0.61) and worse depressive symptoms (r=0.66) and inversely with social (r=-0.50) and physical (r=-0.23) functioning. After 6 months of group exercise, CRL decreased among men who reported experiencing CRL at least some of the time at baseline (p=0.017). Among the whole sample, higher baseline CRL was significantly associated with the greater declines in CRL over 6 months (adjusted-R2=0.16, p< 0.01). A majority of men with prostate cancer may experience cancer-related loneliness, but socially-enhanced group exercise could be an effective countermeasure to improve well-being.

